# Trace Level Arsenic Quantification through Cloud Point Extraction: Application to Biological and Environmental Samples

**DOI:** 10.1100/2012/837672

**Published:** 2012-05-01

**Authors:** Kempahanumakkagari Suresh Kumar, Malingappa Pandurangappa

**Affiliations:** Department of Studies in Chemistry, Bangalore University, Central College Campus, Dr. Ambedkar Veedhi, Bangalore 5600 01, India

## Abstract

A sensitive solvent-free extraction protocol for the quantification of arsenic at trace level has been described. It is based on the reaction of arsenic (V) with molybdate in acidic medium in presence of antimony (III) and ascorbic acid as a reducing agent to form a blue-colored arsenomolybdenum blue complex. The complex has been extracted into surfactant phase using Triton X-114, and its absorbance was measured at 690 nm. The detection limit, working range, and the relative standard deviation were found to be 1 ng mL^−1^, 10–200 ng mL^−1^, and 1.2%, respectively. The effect of common ions was studied, and the method has been applied to determine trace levels of As(III) and As(V) from a variety of samples like environmental, biological, and commercially procured chemicals.

## 1. Introduction

Arsenic is one of the common contaminant of ground water which has been found to adversely affect human health at levels as low as 10 *μ*g L^−1^ [1]. It has a lethal dosage at 50% of the population of 763 mg kg^−1^ of body mass. The maximum contaminant level (MCL) prescribed by the United States Environment Protection Agency (USEPA) for arsenic is 50 *μ*g L^−1^ in drinking water. The World Health Organization (WHO) has recommended MCL for arsenic in drinking water as low as 10 *μ*g L^−1^ [1]. Arsenic is very similar to phosphorous in some physical and chemical properties; that is, the oxides of both elements form colorless and odorless crystalline structures or compounds which are hygroscopic and soluble in water. Due to these similarities, arsenic can often substitute for phosphorous in biological systems [2]. It is well known that arsenic inhibits the key metabolic enzyme pyruvate dehydrogenase and arsenate competes with phosphate for the enzyme which disturbs ATP production and ultimately uncouples oxidative phosphorylation. This inhibition results in the reduction of the energy linked NAD^+^, mitochondrial respiration, and ATP synthesis. The presence of arsenic in the body also increases hydrogen peroxide production which can lead to the formation of reactive oxygen species. Consumption of arsenic contaminated matrices like drinking water, rice, and vegetables lead to various health problems like hyperkeratosis, respiratory, and cardiovascular disorders [3]. Arsenic has been extensively used in several applications mainly in wood preservation, in the production of insecticides, herbicides, drugs, and feed additives, and in poison preparation [4–6]. 

Among the various forms of arsenic, inorganic species like arsenite and arsenate were proved to be more toxic than that of organoarsenicals [3]. Quantification of inorganic arsenic from water samples has been always a challenging task especially at ultratrace level. Instrumental methods like atomic absorption spectrophotometry (AAS), high-performance liquid chromatography (HPLC), and inductively coupled plasma mass spectrophotometry (ICPMS) have been extensively used to quantify this toxic metal ion at trace level [7, 8]. Most of these techniques rely on expensive apparatus, skilled operators, complicated procedures, and time-consuming sample preparation procedures. Hence, spectrophotometric methods find wide spread use in determining metal ions at trace level from a variety of sample matrices due to their easy adaptability even in modestly equipped laboratories. Many of these spectrophotometric methods are less sensitive, and toxic organic solvents like benzene, pyridine, and chloroform were used for analyte extraction [9, 10]. One of these methods requires hydride generation facility which results in the formation of arsenic hydride which is known to be poisonous [11]. Recently, a method has been reported based on microparticle formation of methylene blue dye. The intensity of the color has been quenched by arsenic, and it gave a very low detection limit of 4 ng mL^−1^[12]. Low-cost test kits have been available in the past, but they can be used in higher concentration range, that is, 100–3000 *μ*g L^−1^, which is not sensitive enough to monitor low levels of metal ion in drinking water and other treated industrial effluents. Hence we require inexpensive and sensitive methods for monitoring the arsenic at trace level. Recently, cloud-point-extraction (CPE-) based methods have been extensively used to facilitate preconcentration and separation of the analyte from complex matrices [13].

 Separation and preconcentration of the analyte can be easily achieved by using surfactant in place of organic solvent [13]. The presence of surfactant not only facilitates extraction of analyte efficiently but also enhances the sensitivity of the method [14]. Hence, surfactant-mediated extraction procedures provide very good efficiency in extracting the analyte from a large volume of aqueous solution. This protocol is simple, highly efficient, and less expensive and restricts the use of toxic organic solvents. The present paper describes a simple cloud point extractive determination of arsenic as arsenomolybdenum blue using nonionic surfactant, that is, Triton X-114 at room temperature. The proposed method is simple and sensitive, and it has been successfully applied to determine trace level arsenic from different environmental and biological samples.

## 2. Experimental

### 2.1. Instrumentation

Absorbance measurements were made using a Shimadzu Scanning Spectrophotometer (model UV-3101PC) with 1 cm quartz cuvettes. Calibrated centrifuge tubes with 15 mL volume capacity were used to accelerate the phase separation. All pH measurements were carried out using Control Dynamics digital pH meter (model APX 175). ICPAES analysis was carried out using Jobin Yvon Horiba Spectrometer (model Ultima 2). 

### 2.2. Reagents and Solutions

All chemical reagents used were of Analar grade, and distilled water was used throughout the experiments. Stock arsenate solution (1000 *μ*g mL^−1^) was prepared by dissolving 0.416 g of Na_2_HAsO_4_·7H_2_O AR (SD Fine Chem Ltd., Mumbai, India). Ammonium molybdate solution of 0.015 mol L^−1^ was prepared weekly by dissolving 1.85 g (NH_4_)_6_Mo_7_O_24_·7H_2_O (Merck, Mumbai, India) in 100 mL distilled water and storing in refrigerator. About 0.008 mol L^−1^ of Sb (III) was prepared by dissolving 0.267 g of potassium antimony tartrate (Biddle Sawyer & Co Ltd., Mumbai, India) in 100 mL distilled water. Ascorbic acid solution (SD Fine Chem Ltd., Mumbai, India) of about 0.01 mol L^−1^ was prepared by dissolving 0.176 g in 100 mL distilled water and storing in refrigerator. Sulfuric acid 1.25 mol L^−1^ was prepared by diluting appropriate amount of concentrated acid in cooled distilled water. Triton X-114 (ACROS ORGANICS, NJ, USA) (4% v/v) stock solution was prepared by dissolving 4 mL of concentrated solution in hot distilled water. H_2_O_2_ (30% w/v) (Qualigens, Mumbai, India) was used for sample digestion.

### 2.3. Sample Collection and Preparation

#### 2.3.1. Water Samples

The water samples were collected using polyethylene containers from polluted lake where painted clay idols were immersed after festival procession. The water samples were filtered through Whatman filter paper to remove the suspended particulate matter. Then, 5 mL of the diluted sample was used to determine the arsenic (V) and another 5 mL aliquot of sample was treated with 1 mL each of concentrated nitric acid and H_2_O_2_ for the determination of total arsenic [As(III) + As(V)].

#### 2.3.2. Soil Samples

The soil samples were collected from the agricultural field and soil sludge samples from the pond bed where painted clay idols were immersed. Both samples were collected from the site and stored in polyethylene bags. The soil samples were air dried, and known weight (100 g) of sample was placed in a 250 mL beaker and extracted four times with 5 mL portions of concentrated hydrochloric acid each time. The combined extract was boiled for about 30 min, then the solution was cooled and diluted to 50 mL with distilled water. 5 mL aliquot of diluted sample was used for As(V) determination by the proposed method. Another aliquot of 5 mL was treated with 1 mL each of concentrated nitric acid and H_2_O_2_ solution to determine total arsenic.

#### 2.3.3. Vegetable Samples

The spinach and tomato leaves were collected from local market. They were dried in sun light and grinded into fine powder. 100 g of finely powdered and sieved sample was placed in a beaker. 10 mL each of nitric acid and sulfuric acids were added and heated to 100°C for 20 min, in fume hood. The solutions were cooled, treated with 10 mL of perchloric acid, and heated again in fume hood for 5 min, until the dense fumes of sulphur dioxide disappear completely. Then, solutions were cooled and 1 mL of HCl was added to remove any heavy metal ions present in the sample. The filtered solutions were diluted to 100 mL using distilled water. Then, 5 mL aliquots of diluted samples were used for the estimation of As(V) content as well as total arsenic after treating the sample aliquot with 1 mL each of concentrated nitric acid and hydrogen peroxide.

#### 2.3.4. Biological Samples

**Urine Sample**

Urine samples were collected in sterilized glass containers from male individuals, and 10 mL of sample was diluted to five times. The diluted samples were deproteinated by treating with 2 mL of trichloroacetic acid (30%), and the residue has been removed by centrifugation. The filtrate was treated with 5 mL each of concentrated nitric acid and H_2_O_2_ to oxidize any As(III) present to As(V) in the sample. Then, the solution was diluted to 100 mL and five mL aliquots of diluted samples were subjected for the analysis of arsenic content.

**Nail and Hair Samples**

Hair and Nail samples were collected from adults and washed thoroughly with distilled water followed by acetone and finally dried in an oven at 100°C. About 0.2 g of dried samples were placed in 250 mL beakers separately and, 12 mL of concentrated HNO_3_ followed by 2 mL of HClO_4_ were added. The contents were digested by heating on a sand bath for about 45 min; after the digestion, the solutions were cooled and treated with 5 mL of H_2_O_2_. The reaction mixture was heated again to dryness at 200°C to yield a white residue. Then, 10 mL of 1 mol L^−1^ H_2_SO_4_ was added to the beaker and the contents were heated at 100°C for 1 h and diluted to 50 mL. Five mL aliquots of these solutions were used to estimate the arsenic content.

### 2.4. Chemicals

The commercially procured laboratory chemicals for which the assay has been specified have been used to quantify the arsenic content. One gram of sample was dissolved in water and then treated with 5 mL each of concentrated nitric acid followed by H_2_O_2_. The solutions were diluted to 100 mL, and 5 mL aliquots were used for the analysis of total arsenic content.

### 2.5. Amaranth Dye

1 g of dye sample was dissolved in water and then, treated with 5 mL each of concentrated nitric and H_2_O_2_. The pH of the solution was adjusted to 5 by adding acetate buffer solution and made up to 100 mL. Then, 5 mL aliquot of diluted sample was used for the analysis.

### 2.6. Procedure

Suitable aliquots of arsenate solution (arsenic concentration 10−200 ng mL^−1^) were taken in 10 mL volumetric flasks. Then, 2 mL of 1.25 mol L^−1^ sulfuric acid, 0.2 mL of 0.008 mol L^−1^ antimony (III), 1.2 mL of 0.015 mol L^−1^ ammonium molybdate, and 0.5 mL of 0.01 mol L^−1^ ascorbic acid were added and allowed for 10 minutes for the formation of arsenomolybdenum blue complex. Then, 2 mL of Triton X-114 (4% v/v) has been added and the solutions were diluted to the mark. These solutions were transferred into 30 mL centrifuge tubes and phase separation was achieved by centrifuging them at 3800 rpm for 5 min. The centrifuge tubes were cooled in an ice bath to harden the viscous phase of the surfactant-rich micellar phase. Then, the aqueous phase was separated by simple decantation method. The surfactant-rich micellar phase was homogenized by the addition of ethanol and made up to 5 mL. The absorbance values were measured at 690 nm against the reagent blank.

## 3. Results and Discussion

**Figure 1:**
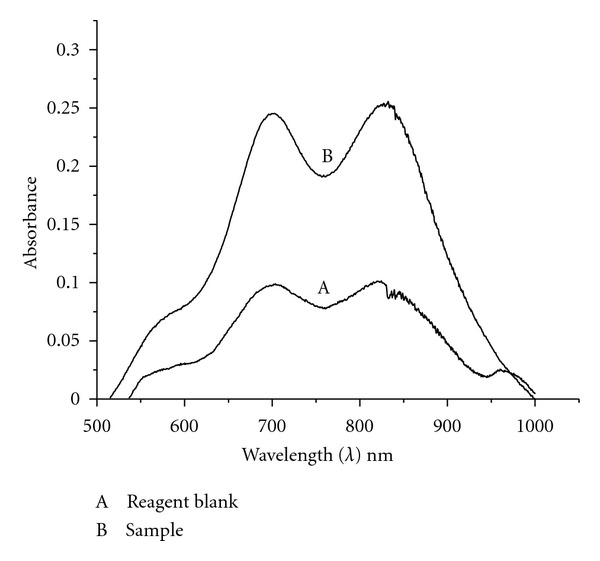
Absorption spectra of arsenomolybdenum blue complex after cloud point extraction.

The proposed method is based on the reaction of arsenic (V) with molybdate to form arsenomolybdate and its reduction to arsenomolybdenum blue complex in presence of a reducing agent. This reaction has been proposed based on the phosphate's reaction with molybdate to form phosphomolybdenum blue in acidic medium and its application to water samples through cloud point extraction [15]. The reaction has been explored to develop a simple and sensitive spectrophotometric method to measure arsenic at nanogram level concentrations. The arsenomolybdate formed in acidic medium with molybdate can be reduced to arsenomolybdenum blue complex with antimony (III) in presence of ascorbic acid as reducing agent. The blue-colored complex exhibited absorption maximum at 840 nm in aqueous condition. Surfactants have been extensively used to sensitize the reaction or to separate the analyte phase without using organic solvent as a medium. Hence, a nonionic surfactant has been used to extract the arsenomolybdenum blue complex by cloud point method at room temperature. The colored complex has exhibited two absorption maxima one at 690 and another at 840 nm in presence of surfactant (Figure 1). The nature of dual absorption maxima of this complex is unknown till now, and the investigations are in progress to find its abnormal behavior. However, the signal to noise ratio is much superior at 690 nm when compared to 840 nm, hence all absorbance measurements have been carried out at 690 nm in the present investigations.

### 3.1. Optimization Study

The initial studies were carried out by extracting the formed arsenomolybdenum blue complex into nonionic surfactants like Triton X-100 and Triton X-114 as the TX series of nonionic surfactants have several advantages over other surfactants like commercial availability, low toxicity, low cloud point formation temperature and high density of the surfactant-rich micellar phase [13]. The quantitative extraction of the complex was obtained by both the surfactants, but, in case of Triton X-100, heating is required for cloud formation but in presence of Triton X-114, cloud formation takes place at room temperature. Hence, Triton X-114 was used as a micellar phase to preconcentrate the analyte species before absorbance measurement. All the parameters influencing the complex formation and cloud point extraction have been optimized.

#### 3.1.1. Effect of Acidity

**Figure 2:**
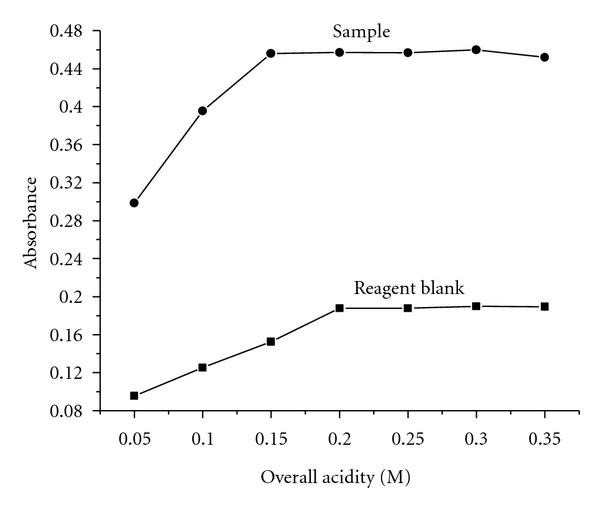
Effect of overall acidity.

The arsenomolybdenum blue complex forms in acidic medium; hence, the effect of sulfuric acid concentration on complex formation has been studied. The higher absorbance values corresponding to the sample versus reagent blank were obtained at an overall acidity value of 0.25 M. The required acidity was achieved by the addition of 2 mL of 1.25 mol L^−1^ sulfuric acid and used in all further studies (Figure 2).

#### 3.1.2. Effect of Surfactant

**Figure 3:**
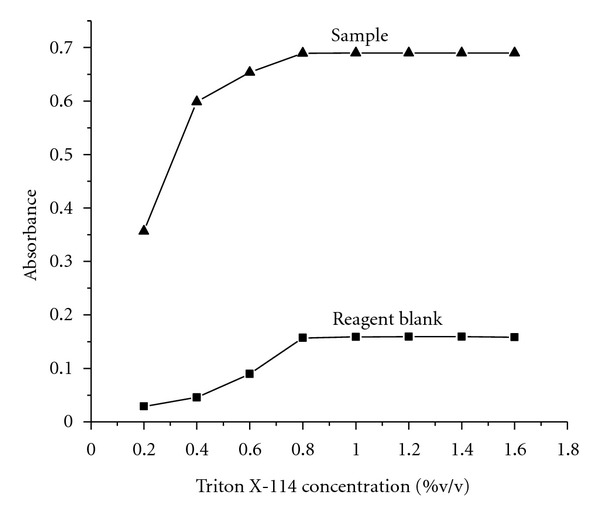
Effect of Triton X-114 concentration.

The effect of surfactant concentration on the quantitative phase separation of analyte through micelle is a crucial parameter in cloud-point-extraction based methods. Hence, we have examined two nonionic surfactants like Triton X-100 and Triton X-114 for the quantitative separation of the complex. Quantitative extraction of the complex from the aqueous phase was obtained by both surfactants, but the extraction of the complex at room temperature was achieved only with Triton X-114. The high density of Triton X-114 facilitates quick phase separation which can be easily achieved by simple centrifugation [16]. In case of Triton X-100, heating is required to attain cloud point temperature whereas Triton X-114 attains clouding at normal condition itself, that is, at room temperature [9]. Hence, Triton X-114 has been selected as a micellar phase for analyte separation. Quantitative extraction of the complex was achieved at 0.8% (v/v). The required surfactant concentration was achieved by the addition of 2 mL of 4% surfactant solution (Figure 3).

#### 3.1.3. Effect of Ammonium Molybdate

**Figure 4:**
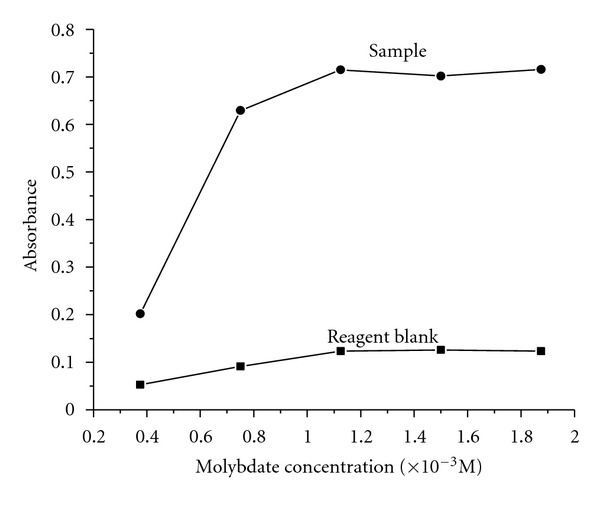
Effect of molybdate concentration.

The effect of ammonium molybdate concentration was carried out in order to get maximum sample absorbance with minimum blank value. The absorbance value of surfactant-rich phase increases with increase in molybdate concentration and remains constant at molybdate concentration beyond 1.2 × 10^−3^ mol L^−1^. Hence, the required concentration was achieved by the addition of 1.2 mL of 0.015 mol L^−1^ molybdate solution (Figure 4). Similarly, the effect of Sb (III) concentration on the complex formation was also studied and the maximum absorbance value for sample was observed at 1 × 10^−3^ mol L^−1^ concentration. It was achieved by adding 0.2 mL of 0.008 mol L^−1^ Sb (III) solution.

#### 3.1.4. Effect of Ascorbic Acid

**Figure 5:**
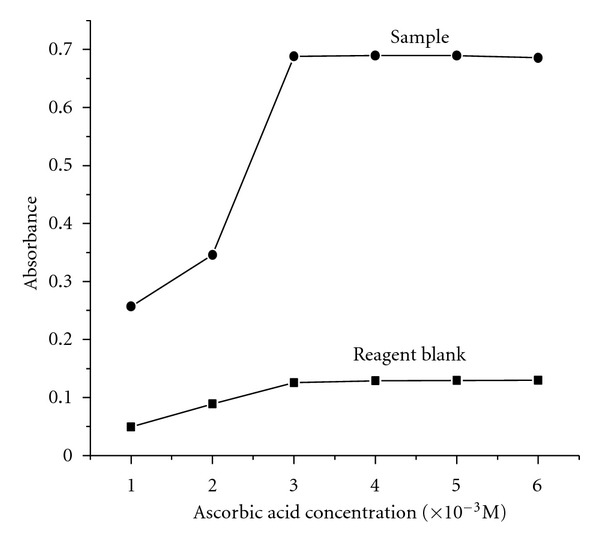
Effect of ascorbic acid.

Various reducing agents like sulfate and ascorbic acid were used to reduce the arsenomolybdate to arsenomolybdenum blue. Ascorbic acid is preferred over sulfate because sulfate is a good reducing agent in neutral condition whereas the complex formation takes place at acidic condition. The optimum concentration of ascorbic acid required for the complex formation has been found to be 4 × 10^−3^ mol L^−1^. The required concentration has been achieved by the addition of 0.4 mL of 0.01 mol L^−1^ (Figure 5).

#### 3.1.5. Effect of Time and Temperature on Cloud Point Extraction

The effects of time and temperature on the cloud point extraction of arsenomolybdenum blue complex from the aqueous phase into micellar phase have been studied. The cloud point formation occurs at room temperature as Triton X-114 cloud point temperature at room temperature. Then, CPE of the complex is going to complete within 10 min, that is, centrifuging the solution for 5 min at 3800 rpm to separate aqueous phase from micellar phase and cooling the separated micellar and aqueous phase in ice bath for 5 min in order to increase the viscosity of the surfactant phase which facilitates easy decantation of aqueous phase from the tube. The separated surfactant phase should be dissolved in suitable organic solvents to decrease the viscosity in order to measure its absorbance value. Various solvents like ethanol, methanol, and acetonitrile were tested. Among these, ethanol has been found to be suitable one because the complex in micellar phase gets homogenized in less volume compared to acetonitrile and methanol. The ethanol-assisted homogenized solution was diluted to 5 mL, and its absorbance was measured at 690 nm against a reagent blank.

### 3.2. Efficiency of Clod Point Extraction

The efficiency of cloud point extraction mainly depends on the hydrophobic nature of the analyte, apparent equilibrium constants in the micellar medium, the kinetics of the complex formation, and the transference between the phases [17]. The arsenate along with molybdate forms arsenomolybdate in acidic condition which on reduction in presence of Sb (III) gives the arsenomolybdenum blue which is hydrophobic in nature. The high hydrophobicity of the complex in water is necessary for preconcentration by cloud point extraction. Under the optimal conditions, the highest extraction efficiency was obtained. The cloud point extraction efficiency increased with the hydrophobicity of the complexes and for the arsenomolybdenum blue complex it is nearly 100%. The extraction efficiency of heteropoly acids like phosphomolybdenum blue and arsenomolybdenum blue (present method) has been found to be 100% because of their hydrophobic nature as well as complete partition due to the efficient binding of these complexes to the micellar phase. Thus, the enhancement factor which has been defined as the concentration ratio of the analyte in the final diluted surfactant rich phase is 24. This enhancement factor facilitates to bring the analyte concentration within the detectable range in the proposed method.

### 3.3. Interference Study

To check the suitability of the proposed method for application studies, the effect of common anions and cations was studied in the determination of arsenic. The anions like Cl^−^, SO_4_^2−^, NO_3_^−^,CO_3_^2−^, F^−^, and citrate did not interfere even at 1000 *μ*g level. However, PO_4_^3−^ and silica interfered positively as they also form heteropoly blue complexes. The phosphate and silicate interference was overcome by treating the sample solution with 1 mL each of 2% calcium nitrate and 3% tartrate, respectively. The cationic species like Ba^2+^, Pb^2+^, Hg^2+^, Al^3+^, and Cu^2+^ form white precipitate which can be removed by centrifuging before adding surfactant. The other cations like Fe^2+^, Ca^2+^, Mg^2+^, Cr^6+^, Zn^2+^, and Ni^2+^ did not interfere even at 1000 *μ*g. This method did not suffer any interference from glucose, citric acid, and amino acids like histidine, and so forth, which are commonly present in the urine samples (Table 1).

### 3.4. Analytical Merits

The analytical merits of the optimized method have been summarized in Table 2. The linear working range of the method has been found to be 10–200 ng mL^−1^. The limit of detection, relative standard deviation, preconcentration factor, and improvement factor of the method were found to be 1.0 ng mL^−1^, 1.4 for 25 ng arsenic, 5 and 24, respectively.

### 3.5. Application Study

In order to check the reliability of the proposed method, it was applied to determine arsenic content in the commercial chemicals where arsenic quantity is certified. The recovery studies were carried out by spiking the biological samples like human urine, human nail, and human hair samples with known quantities of arsenic. These results were found to be compared with the results of ICPAES method which are in good agreement. The arsenic content in surface water, ground water, soil, and vegetable samples were also determined (Tables 3, 4, and 5).

#### 3.5.1. Water Samples

The ground water contamination with arsenic mainly depends on the nature of soil as well as the human activity nearby the region. Arsenic-based paints have been extensively used in painting clay idols throughout the world. These idols were submerged in the lake water or ponds after their procession during the selective festival season in India and some other parts of world. When these clay idols were submerged, the water bodies as well as the soil sledges get contaminated with the arsenic.

#### 3.5.2. Soil Samples

The soil can get contaminated with arsenic by various means. The agricultural soil gets contamination with arsenic by means of manures and agricultural sprays. The soil sludge in our study was collected from the pond beds where painted clays idols were dumped after festivals. These idols slowly dissolve, and pond bed collects clay material containing arsenic.

#### 3.5.3. Vegetable Samples

The plant uptake capacity for arsenic depends mainly on the level of arsenic present in the soil as well as the use of arsenic contaminated water. The arsenic content in spinach leaves and tomato leaves was determined by following the procedure discussed above.

#### 3.5.4. Biological Samples

Arsenic can be measured in human urine, hair, and nail samples to monitor excessive environmental or occupational exposure, to confirm a diagnosis of poisoning in hospitalized victims or to assist in the forensic investigation in case of fatal overdosage. Organic arsenic compounds tend to be eliminated in the urine in unchanged form, while inorganic forms are largely converted to organic arsenic compounds in the body prior to urinary excretion.

## 4. Conclusions

A simple, highly sensitive cloud point extractive spectrophotometric procedure for trace level arsenic quantification in different matrices has been reported. The method is based on the cloud-point-mediated preconcentration of the arsenomolybdenum blue complex and measuring its absorbance. The method can be employed to detect the inorganic arsenic species in various environmental matrices at nanogram levels. This method is much more sensitive than any other spectrophotometric method reported till now including arsenomolybdenum blue method. The use of surfactant in the proposed method is ecofriendly and nontoxic when compared to the conventionally used organic solvents for extraction of the analyte. It provides wide linear range in comparison with some of the reported methods (Table 6). The results obtained by the proposed method have been compared with the ICPAES method, and the measured arsenic levels from different natural samples were found to be in good agreement.

## Figures and Tables

**Table 1:** Effect of foreign ions.

Interferent	Tolerance limit (*μ*g)
Ca^2+^, Cl^−^, Zn^2+^, Ni^2+^, Co^2+^, F^−^	>2000
Cd^2+^, SO_4_^2 −^, I^−^, NO_3_^−^	>1000
Na^+^, K^+^, Mg^2+^, Fe^3+^, Fe^2+^	800
Pb^2+^, Ba^2+^, Cu^2+^, Al^3+^,Hg^2+^	500

PO_4_^3−^	900^a^
	10
	50^b^

SiO_3_^2−^	40
	100^c^

^a^The white precipitate formed by the addition of the above metal ions was removed by centrifuging the solution, and then the reducing agent was added followed by the surfactant for preconcentrating the formed blue complex.

^b^The calcium nitrate was added before adding the molybdate so that phosphate does not form blue complex.

^c^The tartaric acid was used to mask the silica interference, otherwise it forms silicomolybdenum blue and causes positive interference.

**Table 2:** Analytical merits of the proposed method.

Linear working range (ng mL^−1^)	10–200
Limit of detection (ng mL^−1^) (3*σ*, *n* = 5)	1.0
(Relative standard deviation %) (*n* = 5)	1.4
Maximum preconcentration factor	5
Improvement factor	24

**Table 3:** Determination of arsenic from commercially procured chemicals.

Sample	Certified arsenic content (ng)	Arsenic found (ng)	
		Proposed method	ICPAES method
(1) Cupric sulphate^a^ (Analar grade)	5000	4990 ± 24	4990 ± 12
(2) Cupric nitrate^b^ (Analar grade)	1000	990.0 ± 9	1140 ± 18
(3) Sodium hypophosphite Hydrated^c^	4000	4001 ± 16	3800 ± 10
(4) Amaranth dye^d^	3000	2900 ± 12	3000 ± 15

*n* = 5; the values given here are average of five measurements.

^a^Sample was procured from Glaxo Laboratories (India) Ltd., Mumbai with the following certified composition: Cl: 0.003%; As: 0.0005%; Fe: 0.005%; Ni: 0.015%.

^b^Sample was procured from Glaxo Laboratories(India) Ltd., Mumbai with the following certified composition: Cl: 0.001%; Sulphate: 0.0025%; As: 0.0001%; Fe: 0.005%; Ni: 0.01%; Ba: 0.005%; Pd: 0.001%, Bismuth: 0.001%.

^c^Sample was procured from SD Fine Chem Ltd., Mumbai with the following certified composition. As: 0.0004%; Pb: 0.001.

^d^Sample was procured from SD Fine Chem Ltd., Mumbai with the following certified composition. As: 3 ppm; Pb: 10 ppm.

**Table 4:** Determination of arsenic in biological samples.

Sample	Total As (ng)		As(V) added (ng)	Total As(V) found (ng)		Recovery (%)	
	Proposed method	ICPAES method		Proposed method	ICPAES method	Proposed method	ICPAES method
Hair*	ND	ND	20	19.9 ± 1.2	20.0 ± 1.6	99.5	100
Nail*	ND	ND	10	9.2 ± 1.9	9.8 ± 1.1	96.2	98.0
Urine^†^	ND	ND	20	19.6 ± 1.2	20.3 ± 1.2	98.0	101.5

*n* = 5; the values given here are average of five measurements.

ND: Not detected.

*Concentration in ng g^−1^.

^†^Concentration in ng mL^−1^.

**Table 5:** Determination of arsenic in different environmental samples.

Sample	As(V) found in samples	As(III) + As(V) found in samples		As(V) added (ng)	Total arsenic		Recovery (%)	
	Proposed method	Proposed method	ICPAES method		Proposed method	ICPAES method	Proposed method	ICPAES method
Polluted water*	ND	500 ± 12	499 ± 13	—	—	—	—	—
Bore well water*	ND	200 ± 13	200 ± 11	20	220 ± 12	220 ± 12	100	100
Polluted soil^†^	32 ± 2.0	99.0 ± 9.1	98.0 ± 8.3	—	—	—	—	—
Spinach leaves^†^ (Spinacia oleracea)	ND	210 ± 12	209 ± 12	20	230 ± 10	229 ± 12	100	99.5
Tomato leaves^†^ (Lycopersicon esculentum)	ND	500 ± 15	449 ± 11	10	590 ± 12	600 ± 15	98.3	100

*n* = 5; the values given here are average of five measurements.

ND: Not detected.

*Concentration in ng mL^−1^.

^†^Concentration in ng g^−1^.

**Table 6:** Comparison of the proposed method with other methods.

Method	Linear range (ng mL^−1^)	Detection limit (ng mL^−1^)	Preconcentration factor	References
(1) Ion-Pair extraction/spectrophotometry	50–800	—	5.0	[10]
(2) Chemiluminescent method	0–100	0.4	12.5	[11]
(3) Spectrophotometry	0–300	4.0	—	[12]
(4) Cloud point extraction/spectrophotometry	10–200	1.0	5.0	Proposed method
